# Creating molecular macrocycles for anion recognition

**DOI:** 10.3762/bjoc.12.60

**Published:** 2016-03-31

**Authors:** Amar H Flood

**Affiliations:** 1 Department of Chemistry, Indiana University, Bloomington, IN 47405, USA

**Keywords:** anion receptors macrocycles self-assembly surface architectures switches

## Abstract

The creation and functionality of new classes of macrocycles that are shape persistent and can bind anions is described. The genesis of triazolophane macrocycles emerges out of activity surrounding 1,2,3-triazoles made using click chemistry; and the same triazoles are responsible for anion capture. Mistakes made and lessons learnt in anion recognition provide deeper understanding that, together with theory, now provides for computer-aided receptor design. The lessons are acted upon in the creation of two new macrocycles. First, cyanostars are larger and like to capture large anions. Second is tricarb, which also favors large anions but shows a propensity to self-assemble in an orderly and stable manner, laying a foundation for future designs of hierarchical nanostructures.

## Review

“Well, maybe it started that way. As a dream, but doesn’t everything. Those buildings. These lights. This whole city. Somebody had to dream about it first. And maybe that is what I did. I dreamed about coming here, but then I did it.” Roald Dahl, *James and the Giant Peach*

### Early childhood influences

**Figure 1:**
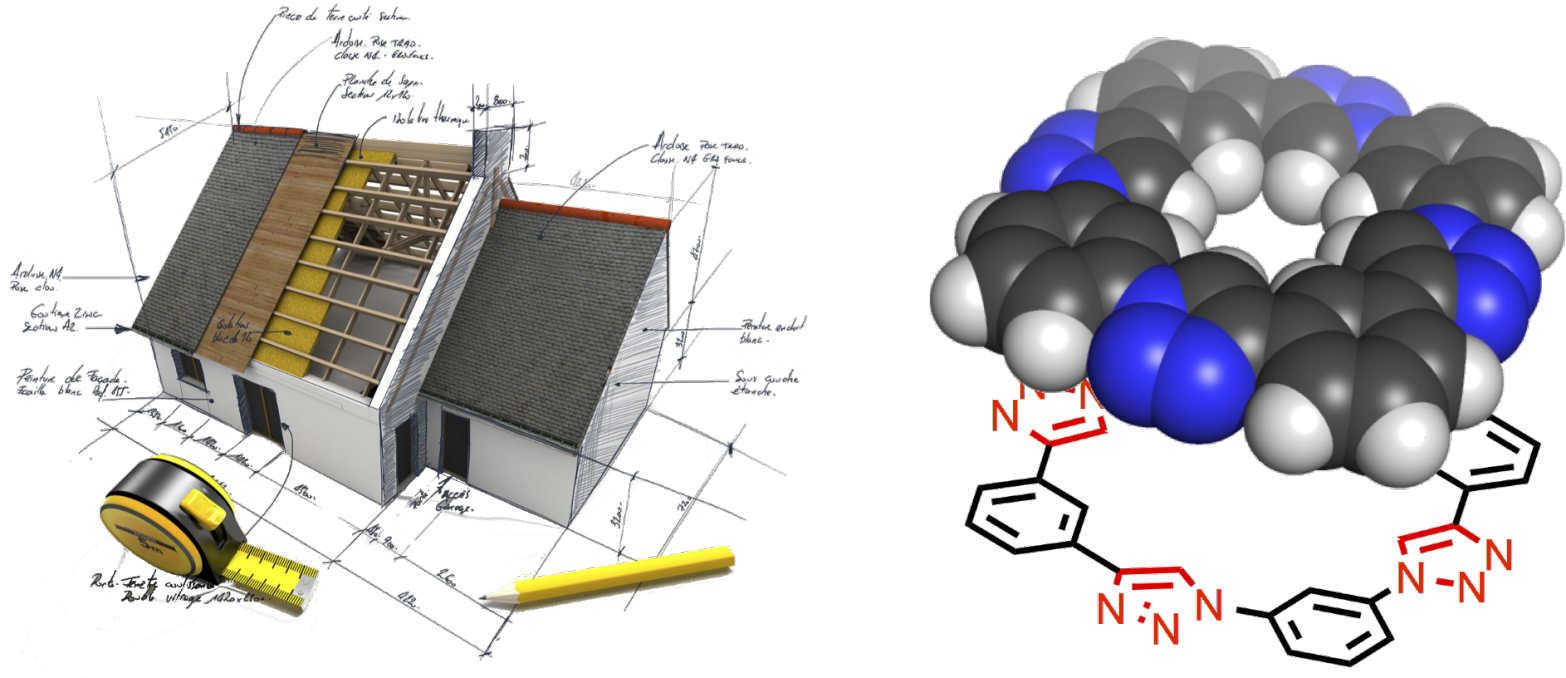
The design and building of a house is just as satisfying as that of a new molecule and often takes the same amount of time (left: Franck Boston copyright 123RF.com).

I was born and raised in Napier, a small town in New Zealand best known as a vacation destination. It was an idyllic place to be brought up where, as kids, we had the freedom to dream. Part of that freedom was born of formative experiences by my father’s side. My father was, and still is, a builder and an outdoorsman. When I was with him, we were either following plans to build a house or making plans to have an adventure. I now recognize that these same skills are used every day in science. In the first case, I learned the satisfaction of making something new (Figure 1), be it a new house or a new molecule. In the second, I learned how to forge a path into unknown territory [1] using only a simple set of core skills; I may have replaced map reading with the scientific method but it involves the same spirit of exploration. I also learned that if I could see the mountaintops on the horizon, I had a fair chance of being able to climb them one day. So it was with my dreams of becoming a scientist.

### Mentors and inspirations

I pursued a bachelor of science at the University of Otago in the city of Dunedin where scholarly life was central to almost everything. I selected chemistry for my major as it provided the most explanatory power of the world around me. I did not start research until my honors year. At the time, I was excited by the research of Keith Gordon and requested to join his group. I worked with Keith on the creation and understanding of metal polypyridyl dyes [2] designed for use in solar cells [3]. I loved being in the laboratory doing research and I continued this work through my Ph.D.

From Keith I learned the importance of designing function into molecules and exploiting them in a homologous series [4] to better extract meaning from their measured properties. I came to appreciate a healthy mix of computation, synthesis and characterization, and I endeavor to use that approach every day. Yet, in spite of all the careful planning, I also learned the importance of simply trying it, or as Keith would say, “suck it and see”.

During my Ph.D., I learned how to be an independent scientist, but I was dreaming of something more, something bigger, and beyond New Zealand’s place at the other end of the world. Looking outward, Fraser Stoddart’s research, in particular, the molecules he made [5] that can be programmed to move and change shape (rotaxanes and catenanes) had hooked my attention. Quite apart from his gift for creating new molecules that are also functional, the number of people who shared co-authorship with him intrigued me.

Joining Professor Stoddart’s laboratory in 2002 as a postdoctoral researcher was an important step in my professional life. I ended up working closely with him, which ultimately provided me with immense perspective. I will admit that it took a long and hard climb from New Zealand into the UCLA group; it was all action and every step took me closer to my mountaintop. I learned everything I could from Fraser: how to run big and small research projects, write scientific papers, and give engaging scientific presentations at conferences. I gained priceless experience. He opened the doors to the world of science that I had only dreamed about during my years at Otago. Being given the chance of independence at Indiana University as an assistant professor was a welcome next step to fulfilling the dream.

### Career accomplishments and highlights

#### Macrocycle discoveries

**Figure 2:**
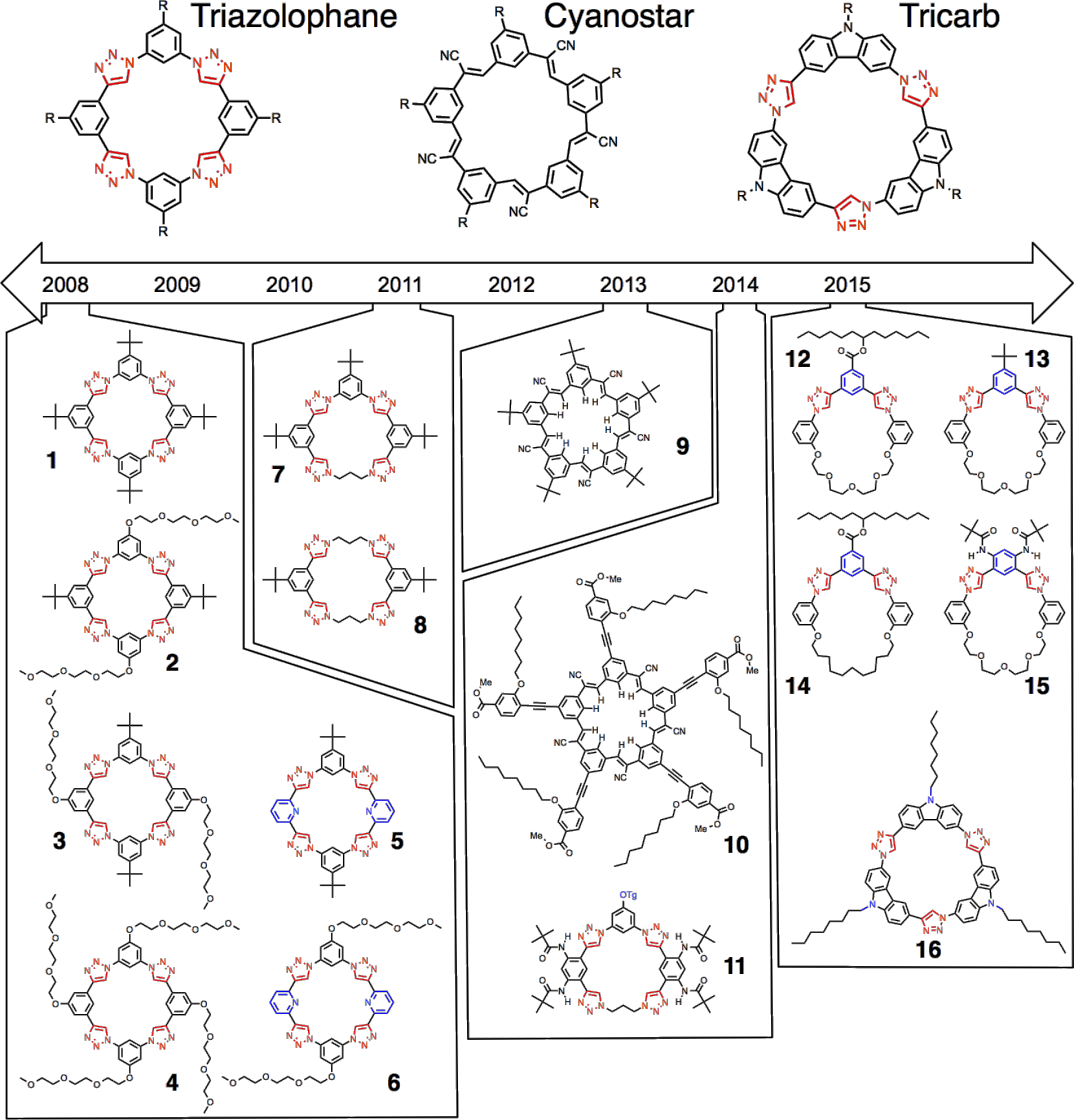
Timeline of anion-binding macrocycles.

Macrocycles have been key to the group’s research findings. The timeline of macrocycle structures (Figure 2) illustrates the depth in triazolophanes [6] and the breadth in the cyanostars [7] and tricarb [8]. Their creative design and their roles in scientific learning will be described in the accounts to follow.

The efforts on macrocycles are particularly rewarding. They are aesthetically and geometrically appealing to work with. In our stables we have triangles, squares and pentagons represented by triacarb, triazolophane and cyanostar macrocycles. The analogy to donut shapes is obvious and the hole in their middles can be both filled with anions and observed using real-space scanning tunneling microscopy (STM) imaging. Their shape persistence makes them ideal for the study of structure–property relationships to enable deep understanding of anion recognition phenomena.

**The identification of 1,2,3-triazoles as linkers, ligands and building blocks.** The synthetic creation of macrocycles sets the scene for the group’s initial and ongoing activities in anion recognition. Triazolophanes [6] (Figure 1) were the first of these macrocycles and the origin of their design warrants description because it represents the first time we made something new.

**Figure 3:**
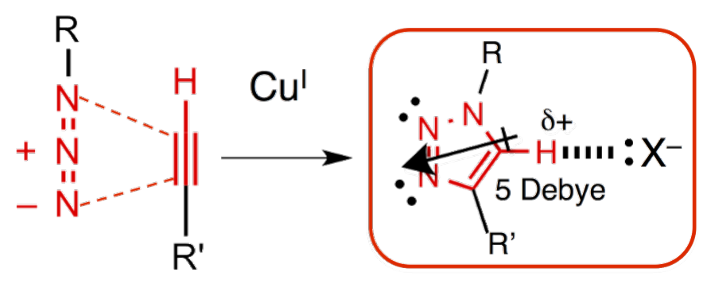
Click chemistry’s copper-catalyzed azide–alkyne cycloaddition (CuAAC) forms 1,2,3-triazoles that stabilize anions by CH hydrogen bonding and ion–dipole interactions.

Triazolophanes were inspired by an overarching philosophy for synthetic design that was derived from Roald Hoffmann’s idea of chemistry being “the same and not the same” [9]. At around this time (2006), click chemistry [10] (Figure 3) had caught my attention for its prevalence but I did not know why it was being used so much. All I could venture was that it was an old reaction, the Huisgen cycloaddition, made good (regioselective) with the aid of copper catalysis. Taking that idea on face value, I reasoned that click chemistry was a new and extremely effective way to make 1,2,3-triazoles. It is not often that either new or newly refined reactions emerge but when they do, they represent plenty of scope for creative chemical design.

At the time, I observed that the triazole linkages were used to bring together two modules, be they a fluorescent tag “clicked” onto a protein or redox-active group “clicked” together with a polymer. But I also thought there must be some latent functionality deriving from the intrinsic structure of the triazole that remained unexplored. It was clear later that Stefan Hecht and Steve Craig had similar thoughts.

**Figure 4:**
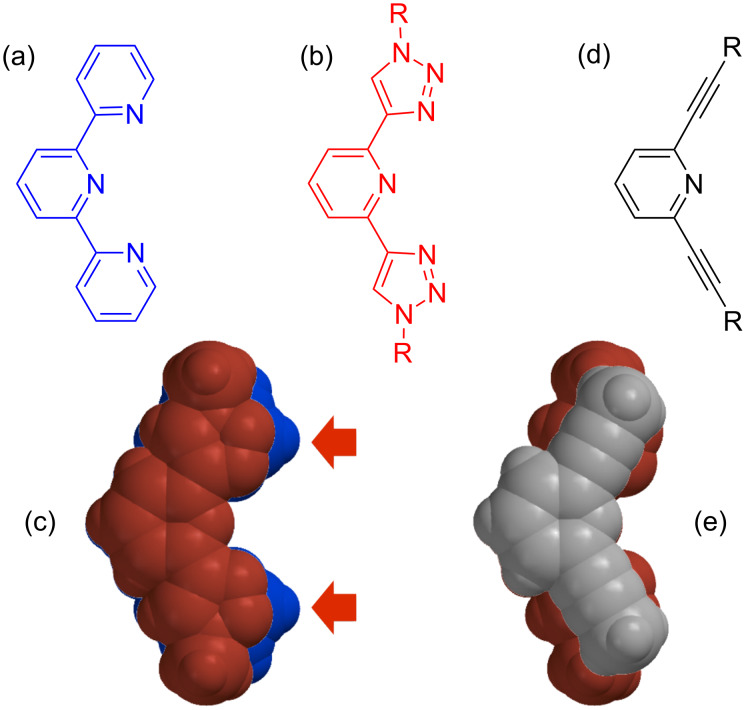
These molecular compounds are the same and not the same.

Invoking Hoffmann’s dictum [9], I originally wondered if the triazoles were the same as pyridines for the purpose of transition-metal coordination, an area in which I received my Ph.D. training with Keith Gordon. I asked my postdoctoral co-worker at the time, Yongjun Li, to assist with testing the idea. We prepared a series of analogs to the common terpyridine ligand (Figure 4a,b). Once made, they indeed bound metals, for example, Fe(II), Ru(II) and Eu(III) [11]. It was at this point that I lifted my head and asked the next critical questions: how are triazoles not the same as pyridines and what does the coordination chemistry teach us about how these 1,2,3-triazoles like to behave?

All our observations indicated that triazoles are sterically small. In the iron complex, we saw the ferrous ion’s preference for triazole change to a water molecule when oxidized up to the harder ferric ion. This process did not occur with the terpyridine control complex. Thus, we reasoned water could easily slip past the triazole nitrogens but not past the pyridine with the steric protection provided by a CH group (see the red arrows in Figure 4c for the overlay of the two ligands). The ruthenium complex showed intermolecular π stacking of ligands on neighboring complexes in the solid state; yet terpyridine analogs did not. Again, having a nitrogen atom in the triazole heterocycle instead of a CH provides less steric interference. Finally, the Eu(III) complex was more hydrolytically stable. With the aid of a crystal structure, we saw the smaller size of the triazoles again circumvented the steric destabilization seen in the CH groups in pyridine that poke uncomfortably into neighboring ligands.

The coordination chemistry taught us that triazoles are sterically small, perhaps small enough to be as innocent as ethynyl units (e.g., Figure 4d and overlay in Figure 4e). If that idea was true, then triazoles became prime candidates for making coplanar structures same as Moore’s [12]. With this realization in mind and a pen in hand, the creation of triazolophanes emerged.

**Figure 5:**
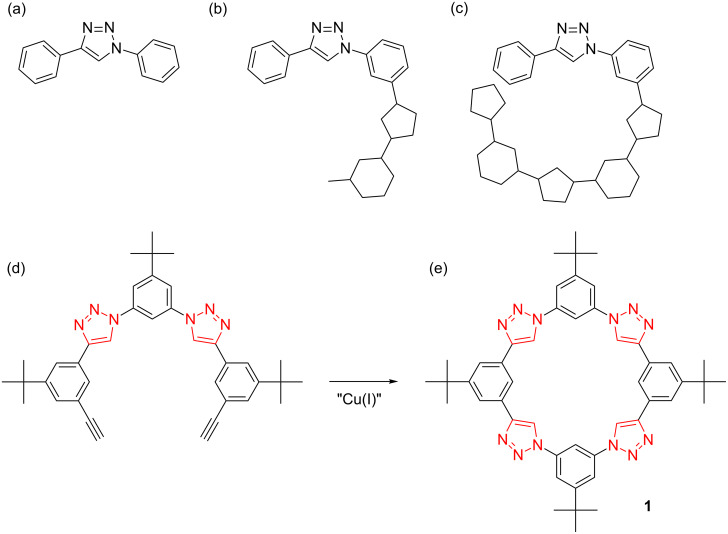
(a, b, c) Sequence of chemical sketches leading to triazolophanes. (d) The precursor that led, by CuAAC, to the (e) macrocycle.

**The creation of triazolophanes.** Sitting in a lecture hall listening to a seminar was the perfect place for doodling molecular designs with triazoles. I started by drawing a triazole and then drew one benzene on either side (Figure 5a). At the time, I was fixated on linear molecules but that was not how this benzene–triazole–benzene triad looked. I recall being bothered that the pentagon shape of the triazole forced me to bend away from linearity; clearly not the same as carbon–carbon triple bonds. But, when I took a calming breath and looked again, I embraced the curvature and just kept on drawing (Figure 5b). Before I really knew what was happening, the alternating benzenes and triazoles quickly and quietly generated a circle resembling a new macrocycle (Figure 5c). I recall being amazed at how well the two ends came together and I thought it was a little too convenient. So, with plenty of time left in the lecture, I carefully redrew the molecular design as a sequence of hexagons and pentagons. I used as much precision as possible and made use of the lines on the notepad for guidance; a hangover from my father’s influence as a builder. If anything, the design got sharper upon redrawing.

A few more things happened that same day to benefit the realization of the macrocycle. I tested the idea using simple molecular mechanics to recapitulate the hand-drawn image with a more realistic model. Later in the day, I asked Yongjun Li, my postdoctoral co-worker at the time, if he thought he could make it, and he said yes. Yongjun and I were fortunate to have built up a working relationship at the time where I would share my latest idea with him and vice versa, then one of us would proceed to knock the idea down. But this time around, the idea fully captured both of our imaginations.

As always, the synthetic pathway leading to a new compound needs to be forged by the traveler making the journey for the first time. While I was happy to see the first few legs pass uneventfully, Yongjun had to pause a long while at the precursor to the macrocycle (Figure 5d). We even considered a few consolation prizes. It is a testament to the perseverance and skills of Yongjun Li that he was able to navigate all the possible dead ends to bring about the first preparation of the macrocycle (Figure 5e). To this day, the stepwise procedure [6] we currently use to make triazolophanes still largely follows the path he set for it.

**Anion binding to triazolophane macrocycles.** At the time when the triazolophane was drawn on the notepad, the central cavity clearly beckoned a guest; after all, nature abhors a vacuum [13]. With hydrogens running around the cavity, it appeared to be predisposed towards anions. However, no one would have anticipated the chloride affinity would be as high as it was.

Triazolophanes bind chloride with an affinity that surpasses many other macrocycles. The affinity that we have come to accept as final for equilibrium **1** + Cl^−^ 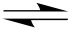 **1**·Cl^–^ is *K*_1_ = 4,700,000 M^−1^ in dichloromethane [14]. This affinity is all the more special and unusual for coming from a receptor that only bears the so-called “weak hydrogen bond” deriving from CH donors [15].

We contend that the triazole CH donors are not weak: the cluster of three nitrogen atoms act together to polarize the CH bond far beyond the intrinsic electronegativity difference of carbon and hydrogen. Added to these four triazoles in the triazolophane are the four benzenes, which also deliver CH donors. Yet, the question of how all these donors perform so well inside the triazolophane defined a research agenda that continues to this day. Ideas such as the macrocycle’s rigid shape-persistence must also play a role.

**Figure 6:**
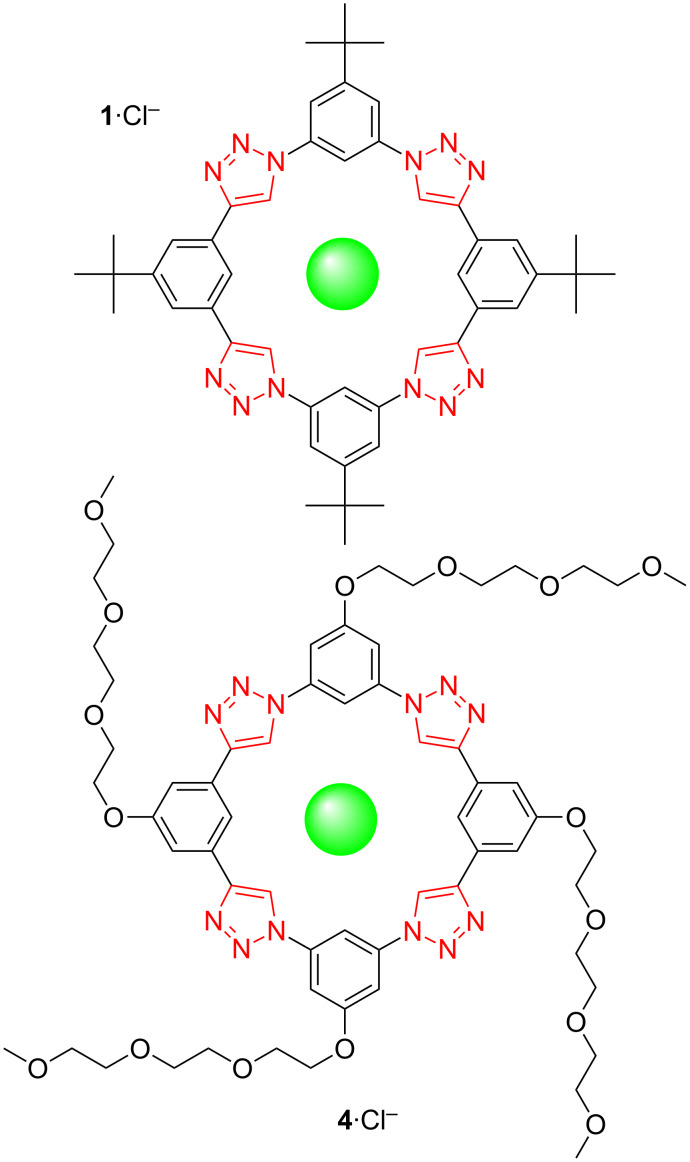
Variation in phenylene substituents weakens chloride affinity from **1** to **4**.

**Testing structure–property relationships for triazolophanes: electronics, halide selectivity, π-stacked sandwich formation, and flexibility.** If the phenylene CH groups were playing a role, we reasoned from Benjamin Hay’s work [16] that electronics would alter the hydrogen bonding strength. We made, characterized, and compared the chloride affinity to a series [17] of triazolophanes with substituents spanning from electron donating *tert*-butyl to more electron donating alkoxys (**1**–**4**) (Figure 2). As expected, the chloride affinity decreased across the series, for example, reducing by a factor of four for **1**·Cl^−^ versus **4**·Cl^−^ (Figure 6). Interestingly, this series allowed us to show that stronger binding occurs in pockets with triazoles linked to the phenylenes through the nitrogen atoms located north and south. We attribute this effect to the electron-withdrawing nitrogen and better angular alignment (Figure 3) of the triazole’s dipoles.

In response to Michael Haley’s question during the first presentation of the triazolophanes in 2007 at Yoshito Tobe’s International Symposium on Novel Aromatics (ISNA-12) on Awaji Island, Japan, we undertook a study of the size selectivity [17]. We found triazolophanes are size-matched to chloride and bromide (*K* ≈ 10^6^ M^−1^) with fluoride being too small (*K* ≈ 10^5^ M^−1^) and iodide (*K* ≈ 10^4^ M^−1^) too large. The binding constants reflect this order. As suggested by Jonathan Sessler in conversations, the bromide is better size-matched but its charge density is just a little less than chloride such that the affinity for chloride is a little higher in dichloromethane. The biggest surprise (and clearest vindication for the rigidity of the triazolophane macrocycle) is the weak fluoride affinity, being an order of magnitude lower than chloride. Typically, charge-dense fluoride produces the largest affinity when the receptor can conform to the fluoride’s smaller size to produce shorter, stronger contacts. Calix[4]pyrroles are a case in point [18]. This conformational rearrangement cannot occur within the triazolophane and so, while the fluoride may have shorter hydrogen bonds, it has fewer contacts than chloride.

**Figure 7:**
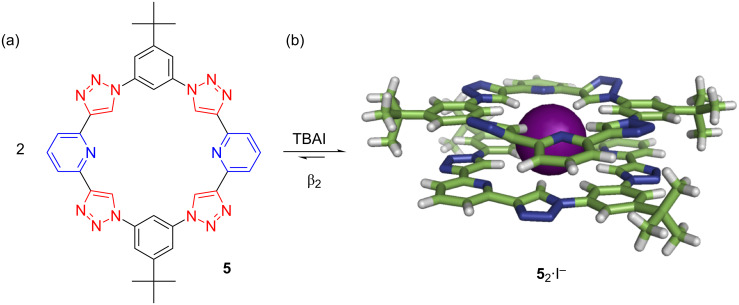
(a) Pyridyl triazolophane and (b) its high-fidelity sandwich around iodide (crystal). Adapted with permission from [20], copyright 2010 Royal Society of Chemistry.

For iodide, it is simply too big for a single triazolophane; so it was a fortuitous and pleasant discovery that, with pyridyl rings [19], 2:1 sandwich complexes form around iodide (Figure 7). In fact, we saw such extreme positive cooperativity that we could not clearly distinguish the 1:1 affinity and reported instead just a fix on the 2:1 stability constant of β_2_ = 8.6 × 10^10^ M^−2^. Interestingly, we observed a rotational offset between the two π-stacked macrocycles that we attributed to the emergence of favorable *anti*-parallel pairing of local dipoles between the two macrocycles.

**Figure 8:**
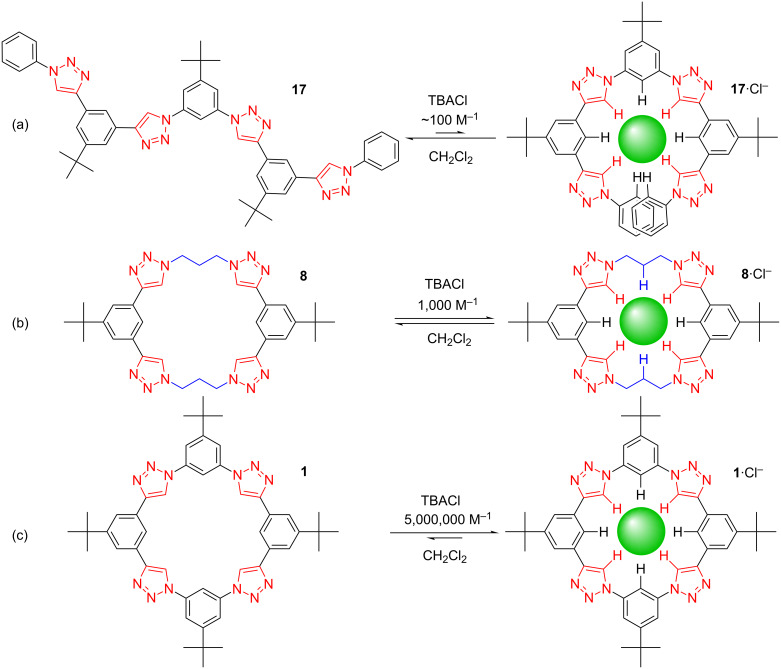
Testing the (a) macrocyclic effect, and (b) effect of rigidity against (c) the parent triazolophane.

The size selectivity of the macrocycles attested both to the triazolophane’s rigidity and that preorganization may be crucial for the large binding affinity. To test this idea, we examined the macrocyclic effect using an oligomer (Figure 8a) that folds up around chloride [17]. The affinity decreased by four orders of magnitude to ≈100 M^−1^, perfectly consistent with expectations. Then, we reduced the rigidity of the macrocycle by replacing two phenylenes with two propylenes (Figure 8b), which decreased the affinity to ≈1,000 M^−1^ [21].

### The affinities we got wrong and our steps to right the wrongs

Side-by-side comparisons of different structures and different anions rely upon the correct estimate of the binding affinity. We originally missed a few features that impacted the accuracy of the binding constants. While these issues are quite common, their impact can be huge: our affinities had to be modified twice from *K*_a_ = 130,000 M^−1^ to 11,000,000 M^−1^ to 4,700,000 M^−1^ [6,14,17]. That is, differences of 100 and then two; factors that have an impact when trying to compare two sets of results.

We had three errors. First, we failed to conduct our titration experiments at concentrations close to the initially determined dissociation concentration, *K*_d_ = 8 μM (= 1/*K*_a_). Our first titrations were conducted at such high concentrations (100 μM) that the moment we added any chloride, it directly converted reactants (macrocycle) into products (1:1 complex). With little to none of the empty macrocycle present after equilibrium was established, the ratio of products to reactants was poorly represented by the observed UV–vis absorption data. We also failed to appreciate that the 2:1 sandwich complexes could also form in solution, a finding we only considered after seeing the 2:1 sandwiches around iodide [19]. What we needed to do was include an additional equilibrium to account for this species.

The third problem with ionic titrations is ion pairing [22], particularly in solvents with dielectric constants (ε) less than 20. Every salt (e.g., tetrabutylammonium chloride (TBACl)) is different and its propensity to be paired in solution should always be evaluated for the concentrations at which the titrations are being conducted. If it is paired, then the receptor has to outcompete the anion from the counter cation in order to be complexed. While ion pairing may be just a little healthy competition, it is too often overlooked because its spectroscopic signature is often hard to detect. Furthermore, once a 1:1 or 2:1 complex has formed, that species is just one big greasy anion and it too can engage with the counter cation. Like the excellent series of papers by Sessler, Gale and Schmidtchen [23–24], we learned all of this the hard way. In their case, when they changed the counter cations, their binding constants were not constant! In our case, when we changed concentrations, the binding constants for the 1:1 triazolophane–chloride complexes were not constant.

**Figure 9:**
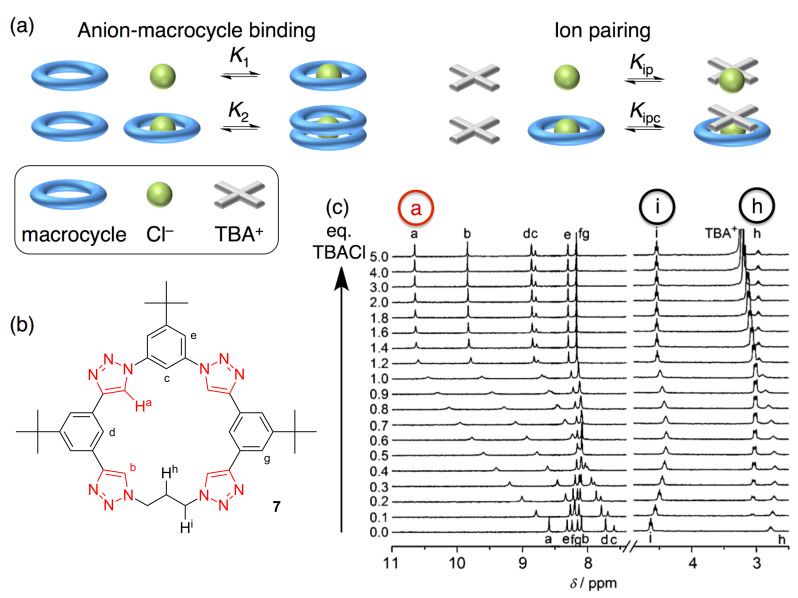
(a) Representations of the four equilibria that dominate in dichloromethane for which the (b) propylene triazolophane’s (c) ^1^H NMR titration data is particularly reflective of the four-equilibrium model. (c) Adapted with permission from [14], copyright 2011 Wiley.

We ended up taming our menagerie of ion pairing and complexation equilibria in a tour de force thermodynamics study [14]. Here I present the full set of four equilibria (Figure 9a) we settled on for the reaction between macrocycle, anion and the TBA^+^ counter cation. Thus, we have the 1:1 species (*K*_1_), formation of the 2:1 sandwich (*K*_2_, β_2_ = *K*_1_ × *K*_2_), formation of the ion pair complex (*K*_ipc_), and competition from ion pairs (*K*_ip_).

As a tribute to the four equilibria and the four species that are formed, the ^1^H NMR titration data for triazolophane **7** is exemplary (Figures 9b,c). Proton H^a^ reflects the 1:1 species by stopping its movements after 1 equiv of chloride is added. Protons H^h^ and H^i^ have inflection points after 0.5 equiv of chloride are added, which is the equivalence point for the 2:1 complex. We observe the ion pairing of the TBA^+^ with the 1:1 complex in the cation’s α proton (≈3 ppm), which has an inflection point at 1 equiv. Consistent with its complexation as a 1:1:1 species, the diffusion NMR signature for the TBA^+^ cation’s α proton showed a lower diffusion coefficient at 1 equiv when it forms the larger ion pair complex MC·Cl^−^·TBA^+^.

Ultimately, any accurate assessment of an equilibrium constant requires inclusion of all the equilibria. There are a few tricks [25]. Some of the less stable species can be diluted out and ion pairing can be avoided by using more polar solvents. Nevertheless, the equilibria operating in solution still need to be assessed. Fortunately, the minor ones can often be omitted. Either their inclusion has a negligible impact on the fitting or they contribute less than 5% to the overall distribution of species in solution such that they are poorly represented in the titration data.

These days, we undertake such detailed multiequilibria analyses about once every graduate student so that they learn how to analyze data accurately. We also make use of more polar solvents that avoid ion pairing. But, in those cases, the triazolophanes then start to self-associate [17,19]. The story of macrocycle self-association is still unfolding in our hands.

None of the multiequilibria fitting would be possible without the use of appropriate software. This allows me to highlight a rewarding collaboration with Douglas Vander Griend who wrote and updates software called SIVVU [26–27], which is the spelling of UVVIS backwards to reflect the idea of the deconvolution of absorbance data into equilibria. In addition, we have benefitted from the use of HypNMR, allowing for the fitting of ^1^H NMR titration data. Both software have their limitations but their usage as tools to unravel complex equilibria is without parallel.

### Cooperativity of ion–pair complexation

**Figure 10:**
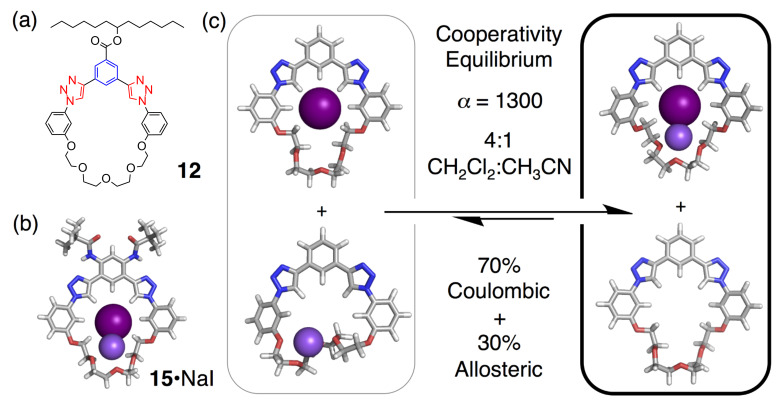
Representations of (a) aryl–triazole–ether macrocycle **12** and (b) the ion-pair crystal structure of **15** with NaI. (c) Cooperativity of ion pairing was revealed using a theoretical assessment of all species. Geometry-optimized structures are shown; note the NaI complex matches the crystal.

The most recent undertaking of ion pairing is to make it a feature and to examine, in glorious detail, how ion pairs can be bound cooperatively inside designed macrocycles [28]. When positive cooperativity emerges, novel selectivity can be engineered [22]. Despite this possibility, we recognized that a deep understanding of ion pairing is still in its infancy. Ultimately, we quantified the cooperativity involved in the salt binding to an aryl–triazole–glycol macrocycle (Figure 2, **12**–**15**, and Figure 10) and examined the cooperativity using theory. We investigated NaClO_4_ and NaI (Figure 10b) experimentally and NaI, NaBr, NaCl theoretically. Theory is able to do things that are impossible in experiment (Figure 10c). As a result, and for the first time, we quantitatively determined that allostery contributed to ≈30% of the cooperativity. The remaining 70% came from Coulombic cooperativity, which dominated the size-dependent trend such that NaCl proved to have greater cooperativity than NaI.

### Using theory for understanding

With accurate values for the 1:1 stabilities of various anion complexes deconvoluted from a veritable stew of other equilibria, quantum chemistry can be used to get an accurate picture of binding. First, we learn how to bring experiment and theory into agreement, then delve deeper into the origins of binding, which also provides a basis for computer-aided receptor design. The fact that triazolophanes are shape-persistent helps immensely for finding agreement between experiment and theory.

**Figure 11:**
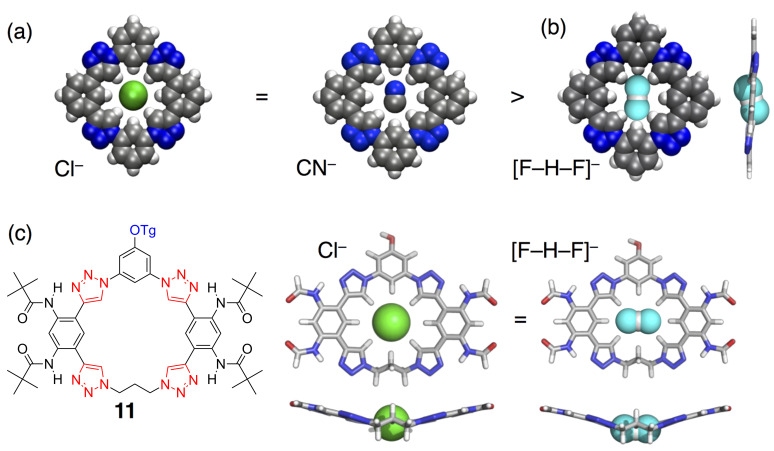
Chloride is used as a comparator for (a) cyanide and (b) biflouride. (c) Computer-aided receptor design was used to optimize the structure to make the chloride’s affinity equal to that of bifluoride. Part (a) adapted with permission from [30], copyright 2011 Wiley. Part (c) adapted with permission from [32], copyright 2014 American Chemical Society.

Working in collaboration with Krishnan Raghavachari, we investigated some of the early ideas about how the triazolophane performs. We confirmed it to be highly preorganized [21,29], that the triazoles are responsible for the majority of the stabilization, and that benzenes offer CH hydrogen bonds at 40% the strength of triazoles. We have examined anions of increasing complexity, ranging across the simple halides, to diatomic cyanide, and triatomic bifluoride. The halides fluoride, chloride and bromide largely follow experiment [29]. However, the cyanide study [30] produced some surprising results. We found in gas-phase calculations and solution-phase experiments that cyanide binds as well as chloride (Figure 11a). Theory helped explain these findings wherein the cyanide rotates in-plane to behave as a pseudospherical anion. In addition, we found nitrogen forms shorter hydrogen bonds [31] even though carbon is the site of covalent bond formation.

### Computer-aided receptor design

We used our collective knowledge to execute our first computer-aided receptor design project [28]. As with the cyanide study, we used the binding of chloride as a yardstick against which to compare and enable understanding of the binding of the triatomic anion bifluoride, [F–H–F]^−^. Unlike cyanide binding, we saw that while chloride and bifluoride had the same affinity in gas-phase calculations, they differed experimentally in solution (Figure 11b). Judging from the calculated geometry, we wagered that the bifluoride anion did not fit very well and that solvent was pulling it out of the binding pocket. We went through a few cycles of computer-aided receptor design to optimize the geometry of the complex. Out targets were to equalize chloride and bifluoride affinities in the gas phase, and to optimize geometries with the anions located in-plane with the macrocycle in order to avoid solvation effects. The hypothesis was that the affinities should also be equal in solution, and after synthesizing the new design, we confirmed the hypothesis (Figure 11c).

#### Cyanostar macrocycles

**Figure 12:**
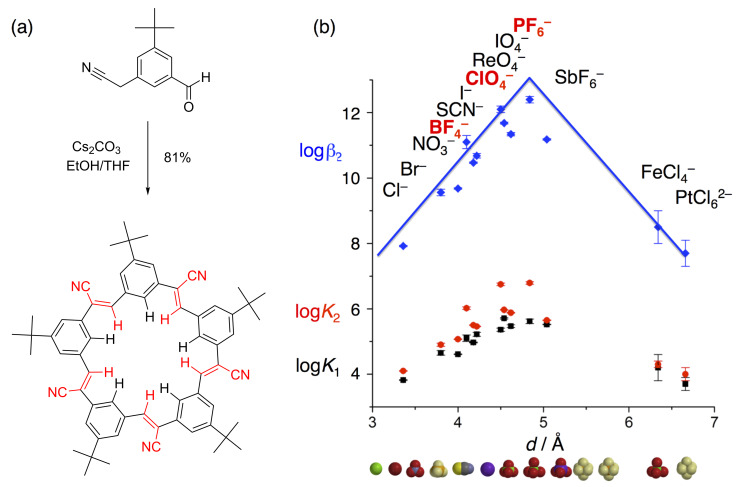
(a) One-pot synthesis of cyanostars. (b) Volcano plot of anion affinities (40:60 methanol/dichloromethane).

Putting all the lessons from the triazolophanes to the test, co-worker Semin Lee designed a wholly new macrocycle, called cyanostar [7] (Figure 12). The motivation for this new work was to generalize the idea of activating CH hydrogen bond donors using electron-withdrawing groups. In cyanostilbene, Lee recognized the cyano group could polarize the CH bond. Following some molecular modeling, he settled on a five-fold symmetric target that demanded a difunctional building block for the Knoevenagel condensation that forms cyanostilbenes. Treating the material to carbonate base-catalyzed conditions, he acquired the product in high yields, now optimized to 80% and scaling nicely up to 10 g quantities.

The effectiveness of the central binding pocket for stabilizing anions was unexpected. Larger than triazolophanes by a whole angstrom, the cyanostar’s cavity was expected to offer weak binding: its cavity was much less electropositive than triazolophanes and its 4.5 Å size was only complementary to anions known to be weakly coordinating [33]. Despite these perceived shortcomings, the affinity towards anions like PF_6_^−^ and ClO_4_^−^ was some 6–9 orders of magnitude higher than most others that had been seen prior [34]. Fortunately, Sindelar’s bambusuril [35] has now been shown to offer similar affinity towards these anions.

**Figure 13:**
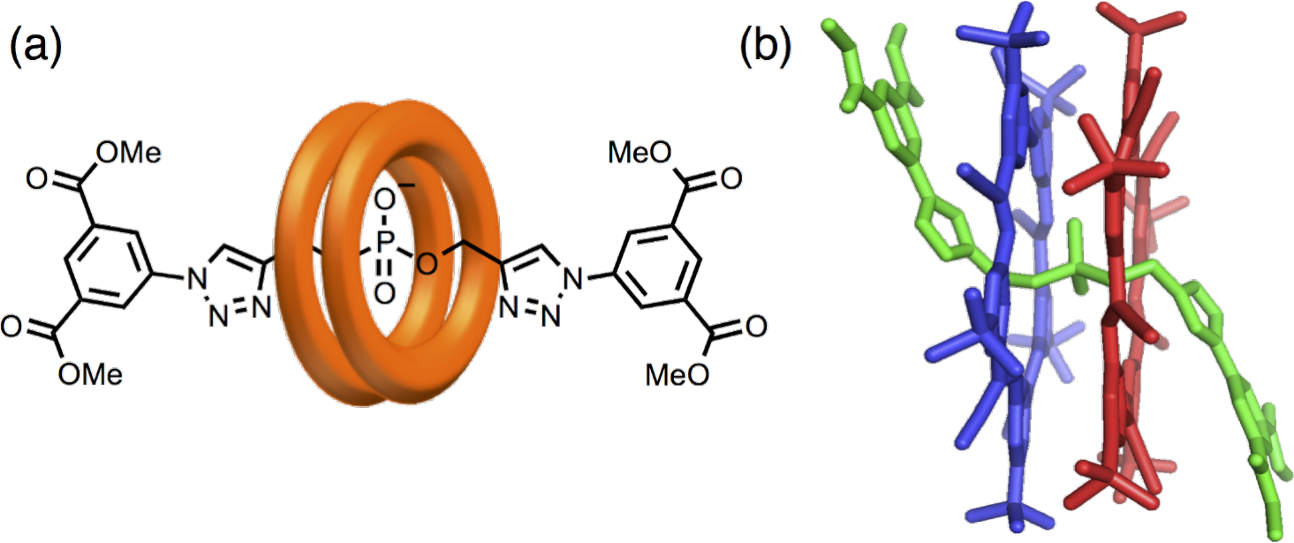
(a) Representation and (b) crystal structure of cyanostar-based [3]rotaxane.

**Figure 14:**
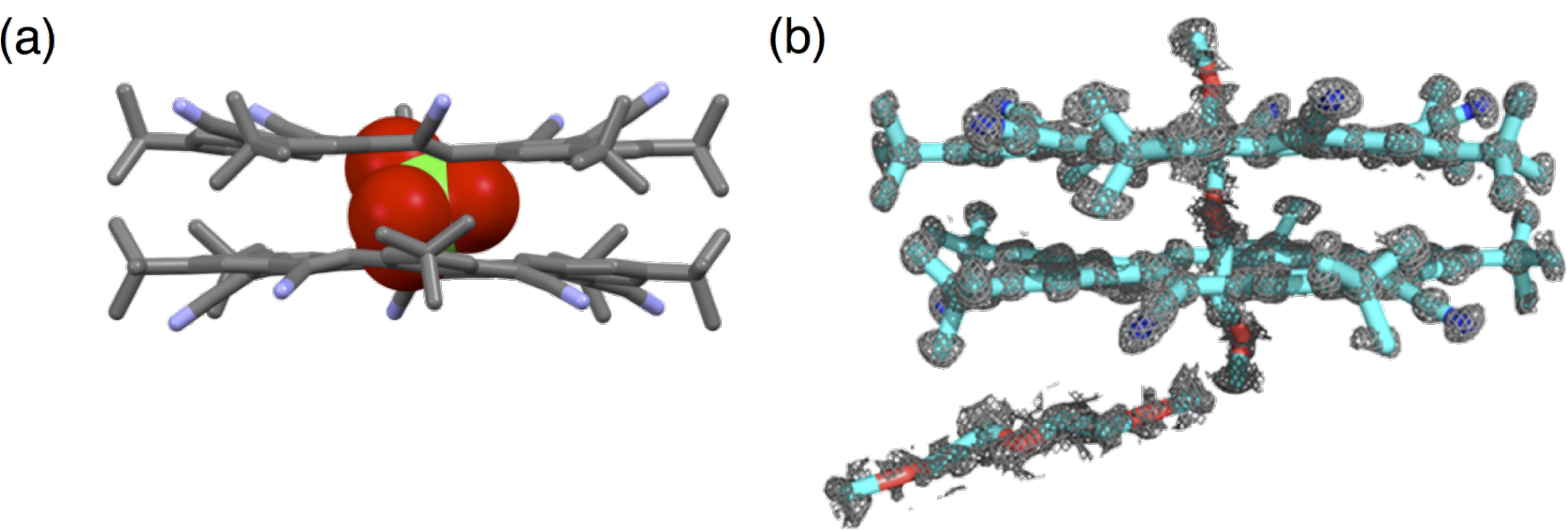
Crystal structures of cyanostar sandwich around (a) perchlorate and (b) diglyme (molecules shown with stick models and representative electron-density contours). Part (a) reproduced with permission from [36], Copyright 2014 Royal Society of Chemistry; Part (b) adapted with permission from [37], copyright 2015 American Chemical Society.

The performance of the cyanostar macrocycles is still being explored. The large binding pockets and the *C*_5_ symmetry provide a basis for a lot of new chemistry. This includes use of phosphodiesters as templates for the synthesis of [3]rotaxanes [7] (Figure 13). The other aspect of these macrocycles arising from their π surface is their propensity to stack. This can be seen in the crystalline phase with dimer formation either around anionic guests like perchlorate [36] (Figure 14a) or, in the absence of an anion, around adventitious solvents of crystallization, like diglyme (Figure 14b) [37] .

**Figure 15:**
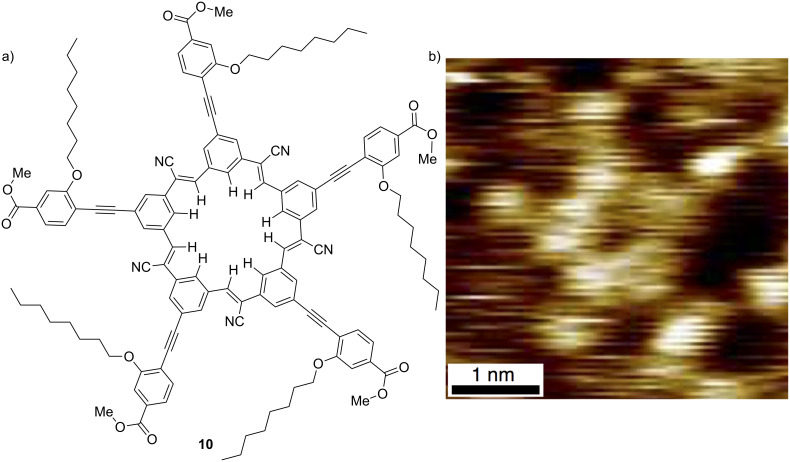
(a) Star-extended cyanostar and an (b) STM image cropped from a 2D lamellar lattice. Part (b) adapted with permission from [36], copyright 2014 Royal Society of Chemistry.

The π-surface was enhanced by extending the cyanostar’s extremities (Figure 15a) in a bid to program the molecule’s self-assembly into a 2D array on graphite [36]. This work was undertaken in collaboration with Steve Tait who is able to resolve molecules using STM. It was gratifying to see the molecules assemble and even more so to observe (Figure 15b) the macrocycle’s star shape.

#### Tricarbazolo triazolophane macrocycles

**Figure 16:**
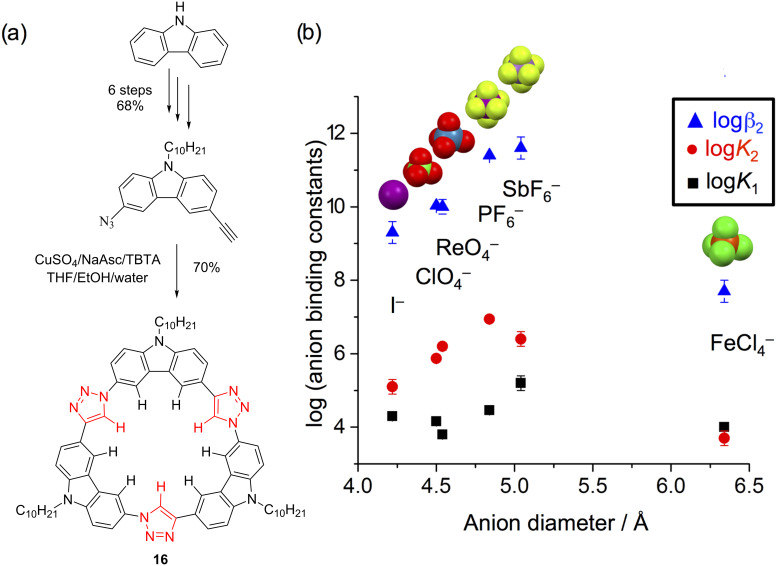
(a) Synthesis and one-pot macrocyclization of the tricarb macrocycle. (b) Volcano plot of anion affinities (20:80 methanol/chloroform). Part (b) adapted with permission from [8], copyright 2016 Wiley.

Perhaps co-worker Semin Lee had acquired a taste for discovery because he created another class of macrocycle. The tricarbazolo triazolophanes [8] (tricarb for short, Figure 16a) were designed to be made in one pot using click chemistry and to prepare another potent binding pocket for anions composed solely of CH hydrogen bond donors. Now just a little larger than cyanostars at 4.8 Å, their affinities showed peak affinity for slightly larger anions (Figure 16b). The tricarb structure is highly planar and this is where its properties begin to depart from cyanostar’s. This planarity leads to extremely high self-association behavior. While a crystal structure has so far been elusive, the pattern of molecular packing has been examined from studies of surface assembly.

**Figure 17:**
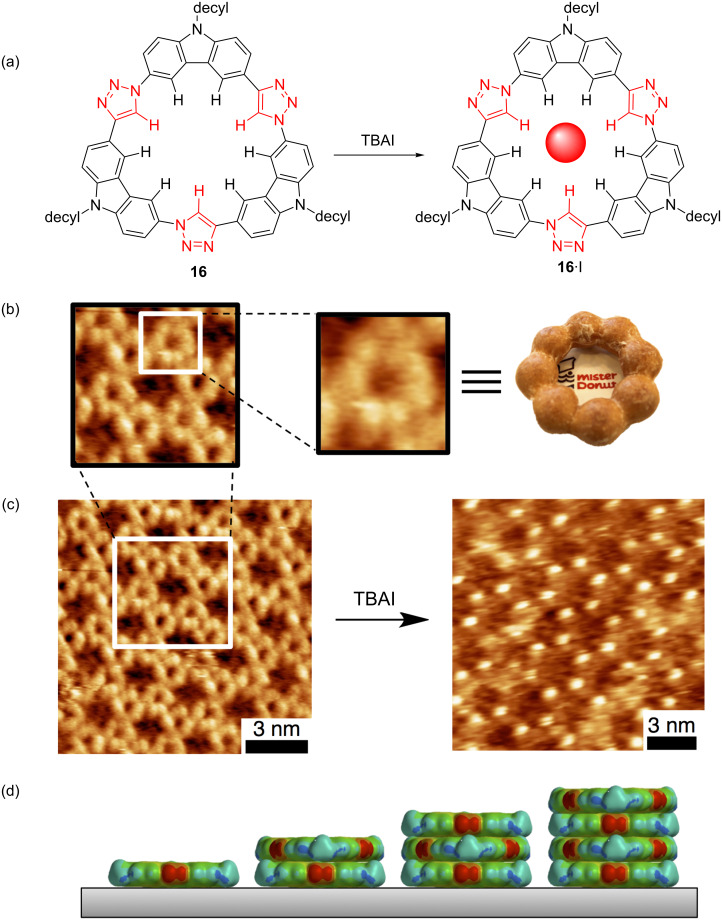
(a) Tricarb binds iodide. (b) Tricarb’s single-molecule STM image resembles a donut. (c) Honeycomb surface patterning of tricarb before and after binding iodide. (d) Representations of how tricarb stacks on graphite. Parts (b) and (c) adapted with permission from [8], copyright 2016 Wiley.

We inspected the tricarb macrocycle’s surface self-assembly and anion binding (Figure 17a) using STM and were surprised by what we saw. Clear in the imaging (Figure 17b) were their shapes, looking like lumpy donuts; a shape similar to the one offered by Japan’s Mister Donut. Interestingly, these macrocycles displayed a reliable propensity for clean self-association into 2D patterns of fused rosettes (Figure 17c). This pattern allowed us to readily discern binding of iodide anions as bright features constituting a pattern with the same unit-cell dimensions as the parent honeycomb. These macrocycles had another characteristic that was unexpected; they showed concentration-driven stacking directly on top of one other into uneven multilayered ultra-thin films (Figure 17d) about three to four layers thick. This layering is unusual for such self-assemblies and, thus, this is a fruitful area for us to investigate further by considering how to program the outsides of macrocycles to direct 3D packing.

#### Crescent receptors

**Figure 18:**
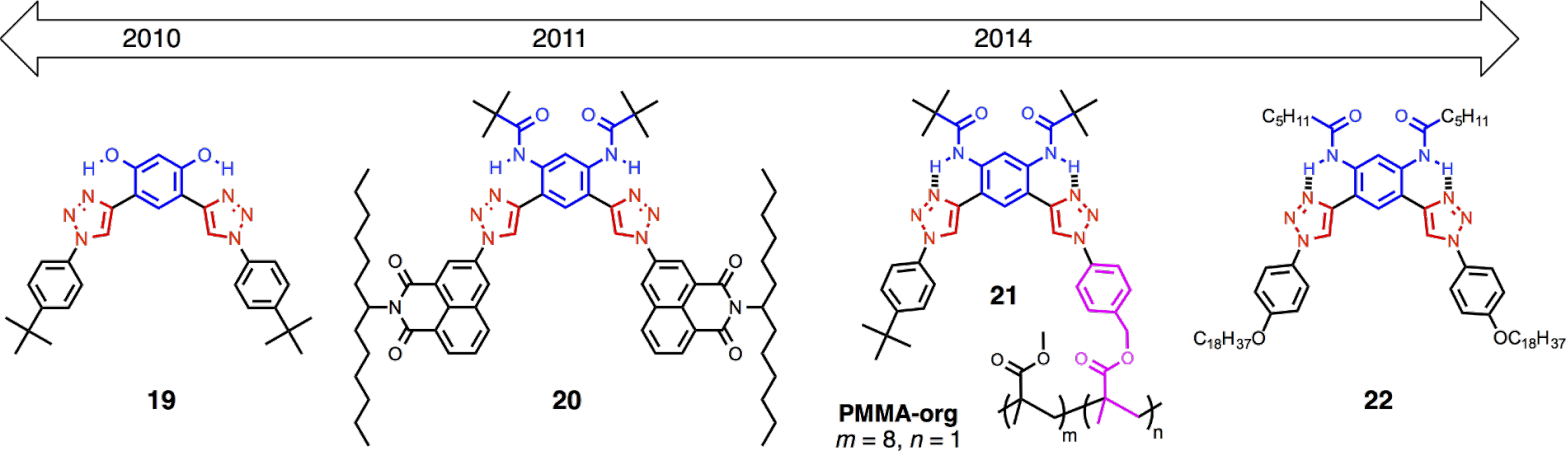
Timeline of crescent-shaped anion receptors.

Crescent receptors, while not as elegant as macrocycles, are succinct models for testing ideas. Over the years (Figure 18), we have tested intramolecular hydrogen bonding for preorganization (**19**) [38], new CH hydrogen bonding from naphthalimides (**20**) [39], and investigated the transfer of function to polymeric constructs (**21**) [40]. The most recent crescents (**22**) provided a basis to investigate surface self-assembly [41], anion binding and switching at interfaces [42]. They also provide a base for foldamers; a series of crescents linked together into oligomers with interesting dynamic shapes.

#### Foldameric anion receptors

**Figure 19:**
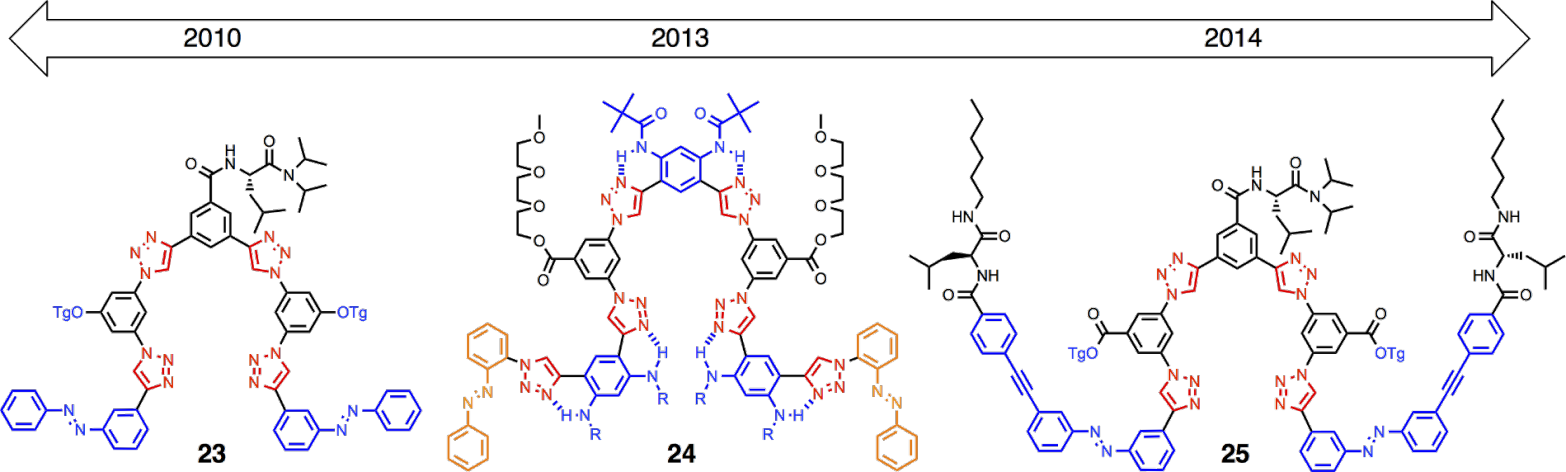
Timeline of anion-binding foldamers.

Complementing our creation and study of macrocycles is work on foldamers (Figure 19). With their flexibility, foldamers are excellent “character foils” to rigid macrocycles. As a class of compounds, foldamers [43] are highly modular and thus benefit greatly from the prevalence of azido and alkynyl building blocks that serve as precursors to click chemistry. Our research program is bioinspired [44] for the control of anions for separation purposes. We have utilized them for regulating chloride [45]. Starting with an on–off binding and release ratio of about 10 with foldamer **23** [46], and, by using secondary contacts often seen in biology, we have reached up to a ratio of 84 with **25** [47]. We also saw protein-like driving forces elicit unprecedented Cl^−^ binding in semi-aqueous solutions (50:50 water/acetonitrile). The aryl-triazole foldamer **24** formed a double helix with overall stability of β_2_ = 10^12^ M^−2^ [48]. Importantly, while we observed clear penalties to the binding affinity when we added extra water, we also saw that extra water increases the stability of the duplex. The analogy to hydrophobic collapse in proteins is clear.

#### Other passions, interests, and activities

Outside of research, my other passions are family, mountain biking, and visits to New Zealand and Japan. The family is young and the mountain biking is new, so finding the right balance with research is critical and rewarding.

**Figure 20:**
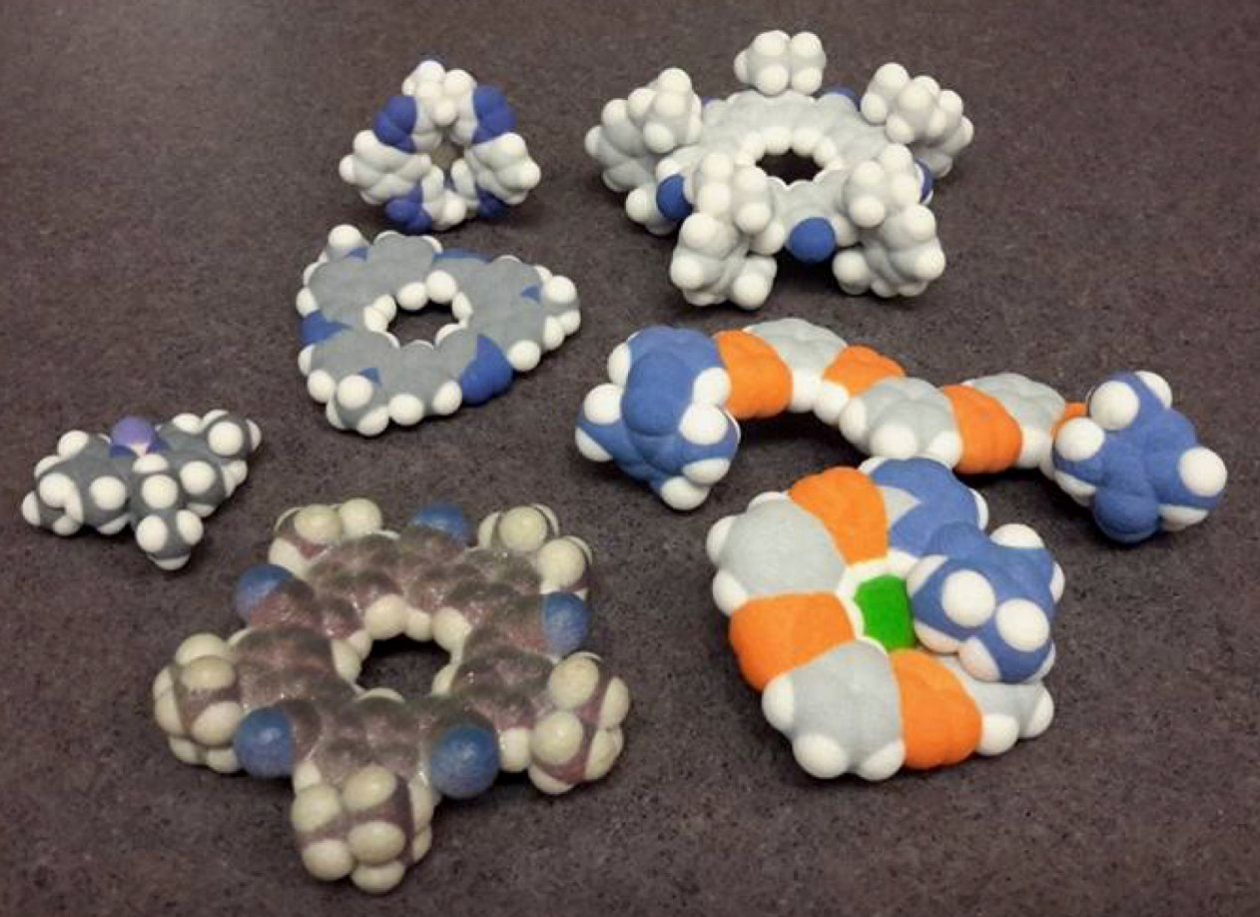
Family portrait of 3D-printed molecular receptors.

My group and I are also enjoying 3D printing of molecules (Figure 20), an activity that Ognjen Miljanic and I recently described [49]. Having early access to a full-color 3D printer in Indiana University’s School of Fine Arts helped my early adoption of holding and seeing molecules. Now we use it for “image training” (thanks to Makoto Fujita for the turn of phrase) as much as to show off the shape and function of molecules.

### Future perspective

There are a few grand challenges inspiring us going forward. Underpinning each of them is a central philosophy: that the chemical nature of all matter motivates us to consider how we might design molecules to have an impact on the world of human experience. The anion recognition work has over-arching implications across a broad swath of modern life, from human biology, to the environment, to industry. The opportunity for the use of molecules in separations and environmental remediation is of continuing importance to us. The ongoing effort to plumb the depths of anion recognition synergizes with this goal, and it also brings computer-aided receptor design into focus as a very real prospective for the future [32].

We have recently been inspired to program the surfaces of molecules to direct their hierarchical assembly into molecular materials. The discovery of tricarb’s 3D assembly at interfaces [8] is igniting this work. Our inclination to collaborate with theorists also opens up the chance to design those hierarchical assemblies using computer-aided design. The long-term ramifications include the ability to create semiconductor structures by simple self-assembly. Thus, the goal is to contribute scientific understanding on how to program the nanostructures of advanced technology by employing bottom-up molecular design.
